# Multiple quasicrystal approximants with the same lattice parameters in Al-Cr-Fe-Si alloys

**DOI:** 10.1038/srep40510

**Published:** 2017-01-13

**Authors:** Zhanbing He, Hua Li, Haikun Ma, Guowu Li

**Affiliations:** 1 State Key Laboratory for Advanced Metals and Materials, University of Science & Technology Beijing, Beijing 100083, China; 2 Crystal Structure Laboratory and National Laboratory of Mineral Materials, China University of Geosciences (Beijing), Beijing 100083, China

## Abstract

By means of atomic-resolution high-angle annular dark-field scanning transmission electron microscopy, we found three types of giant approximants of decagonal quasicrystal in Al-Cr-Fe-Si alloys, where each type contains several structural variants possessing the same lattice parameters but different crystal structures. The projected structures of these approximants along the pseudo-tenfold direction were described using substructural blocks. Furthermore, the structural relationship and the plane crystallographic groups in the (a, c) plan of these structural variants was also discussed. The diversity of quasicrystal approximants with the same lattice parameters was shown to be closely related to the variety of shield-like tiles and their tiling patterns.

As one kind of structurally complex alloy phases, quasicrystal approximants have triggered wide interest owing to the challenge in solving their complex crystal structures and their possibly exceptional properties^1^,^2^,^3^. The substructures or the structural tiles of approximants are the same as those of the corresponding quasicrystals, but are arranged periodically in approximants while a quasiperiodic arrangement is observed in quasicrystals. Therefore, understanding the crystal structures of the approximants is beneficial for revealing the crystal structures of the quasicrystals, which are much more complicated than approximants because of their lack of translational symmetry.

X-ray single crystal diffraction is the most popular technique for structure determination, and the crystal structures of some approximants have already been solved by this technique^4^,^5^,^6^,^7^,^8^,^9^,^10^,^11^. Generally, one will preliminarily consider whether the phase is new or known according to the lattice parameters, which are determined preferentially by X-ray single crystal diffraction before collection of the precise data of the diffraction spots. Prior to the structure determination, the phases with the same lattice parameters are usually considered to be the same if the single crystals for X-ray diffraction are obtained from samples with the same compositions and preparation conditions.

Compared with X-ray single crystal diffraction, transmission electron microscopy (TEM) is more widely used to understand the crystal structures of approximants, because it is difficult to achieve large and high-quality single crystals for X-ray single-crystal diffraction. For example, the structural models of some approximants were proposed by electron crystallography^12^,^13^,^14^,^15^,^16^, based on the structural relationships of the known and unknown approximants determined from the high resolution transmission electron microscopy (HRTEM) images. Some typical structural blocks, such as hexagon (H), boat (B), star (S), and decagon (D)^17^,^18^,^19^,^20^, are often found in Al-based decagonal quasicrystals (DQCs) and their approximants; and the rich combinations of these substructures lead to an abundance of approximants^21^,^22^,^23^,^24^,^25^,^26^. Among them, the approximants with different lattice parameters may consist of the same structural blocks, for example, the H tile for (1/0, 2/1) and (1/1, 1/1) approximants^27^,^28^. On the other side, the approximants of Al-based DQCs with the same/similar lattice parameters in different alloy systems, for example, the O_1_ approximants in Al-Mn-Ni^29^ and Al-Cu-Fe-Cr^21^, were found to have the same the structural blocks and tiling patterns.

Recently, we found that the orthorhombic (3/2, 2/1) phase, previously reported in Al-Mn-Ni^29^,^30^ (named as O_1_, with *a* = 3.27 nm, *b* = 1.25 nm, and *c* = 2.38 nm in ref. 29), Al-Mn-Pd^31^, Al-Cu-Fe-Cr^32^, and Al-Fe-Cr^33^, has an additional type of structural tiling in the Al-Cr-Fe-Si alloys^34^, which is different from the known structural tiling in Al-Mn-Ni^29^. Herein, we report a series of approximants with the same lattice parameters but different crystal structures in Al-Cr-Fe-Si alloys, based on the experimental results of the high-angle annular dark-field (HAADF) scanning transmission electron microscopy (STEM) images at an atomic resolution. Note that the same lattice parameters mentioned here are based on the assumption that the structural tiles of the approximants are perfect, and therefore, they exhibit no distortion. Accordingly, the same structural tile has the same shape and size. For clarity, we adopt (*F*_*n*_/*F*_*n*−1_, *F*_*m*_*/F*_*m*−1_) to name the Fibonacci approximants, where *F*_*n*_ and *F*_*n*−1_, as well as *F*_*m*_ and *F*_*m*−1_, are neighboring numbers in the Fibonacci sequence^35^. Furthermore, we use (*F*_*n*_/*F*_*n*−1_*, F*_*m*_/*F*_*m*−1_)_*x*_, with *x* = 1, 2, 3 … to distinguish the approximants with the same lattice parameters but different crystal structures. Consequently, the lattice parameters in the pseudo-tenfold plane of the Fibonacci approximants are intuitively reflected by the number of *F*_*n*_ and *F*_*m*_^29^,^35^.

## Results and Discussion

### (2/1, 3/2) approximants

**Figure 1:**
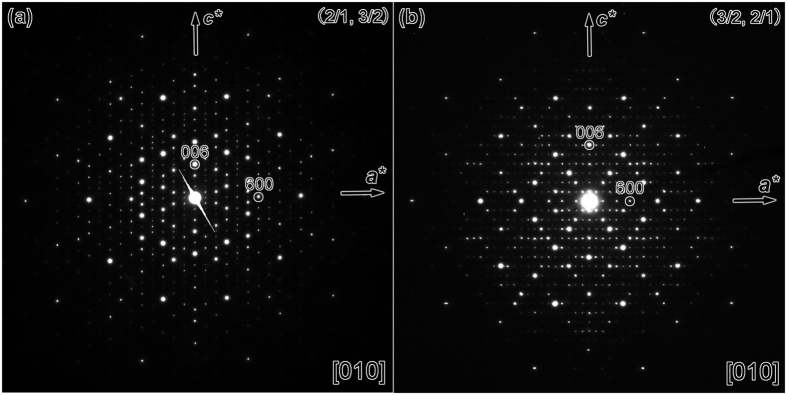
Selected-area electron diffraction patterns along the [010] zone axis of the two primitive Fibonacci approximants. (**a**) (2/1, 3/2) (from Sample 1), (**b**) (3/2, 2/1) (from Sample 2).

Besides the *B*-centered (3/2, 2/1) approximant in the Al-Cr-Fe-Si system^34^, we observe two additional primitive Fibonacci approximants in the Al-Cr-Fe-Si system by selected-area electron diffraction patterns (EDPs), as shown in Fig. 1. The unit cell, measured from Fig. 1a, is calculated as *a* ≈ 2.0 nm and *c* ≈ 3.8 nm, close to those of the (2/1, 3/2) Fibonacci approximant (*a* = 1.99 nm, *c* = 3.79 nm)^29^, and thus was ascribed to the (2/1, 3/2) type. The parameters of *a* and *c* of the second primitive Fibonacci approximant in Fig. 1b is calculated as *a* ≈ 3.2 nm and *c* ≈ 2.3 nm (from the selected-area EDP), corresponding to the (3/2, 2/1) approximant, which was reported previously in Al-Mn-Ni alloys^29^,^30^. The composition of the (2/1, 3/2) approximant in Fig. 1a is measured as Al_54.9_ Cr_22.5_ Fe_9.6_ Si_13.0_, and that of the (3/2, 2/1)-type approximant in Fig. 1b is Al_52.7_ Cr_23.1_ Fe_8.3_ Si_15.9_, implying that their compositions are very similar.

**Figure 2:**
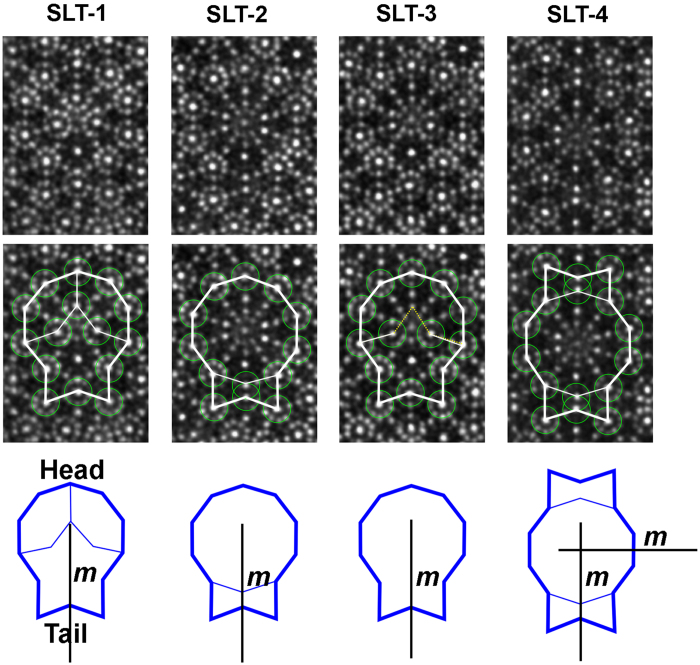
Four kinds of shield-like tiles (SLTs). The first row: HAADF-STEM images at an atomic resolution. The second row: the outlines of four SLTs depicted by the thick white lines. The third row: schematic diagram of four SLTs. Note that the symmetric element of the mirror is indicated by *m*.

A shield-like tile (SLT) was used to described the crystal structures of the (3/2, 2/1) and (2/1, 3/2)-type approximants, showing the advantage of concision^34^. Furthermore, three additional kinds of SLT structural blocks were observed in the Al-Cr-Fe-Si system in this report besides that adopted previously^34^,^36^. Therefore, the different SLTs were renamed as SLT-1, SLT-2, SLT-3, and SLT-4 to distinguish between them (Fig. 2). We describe these SLTs preferentially because the structural tiling patterns of the approximants in this paper will be mainly analyzed according to the SLTs.

The atomic resolution structural images of these SLTs are shown in the first row of Fig. 2, and their structural characteristics are demonstrated by both the white lines and the small green circles in the second row, where the small green circles represent the smallest D clusters located on the vertexes of the S, H, and SLT. The SLT-1 tile consists of two H tiles and one S tile, which was actually the reported SLT in refs 34 and 36. Neither perfect S nor H tiles (where each vertex of the perfect tiles should have the same structural configuration) could be deduced from the SLT-2 from the structural point of view, because the head of SLT-2 was quite similar to the corresponding part of the perfect D cluster in the Al-Cr-Fe-Si system^36^. Therefore, the SLT-2 could be decomposed into one decagon and one bowtie (BT) tile in geometry, rather than a combination of H and S in the SLT-1. We observed that the SLT-1 and SLT-3 are quite similar. However, the smaller D cluster was missing on one vertex of the S tile in the inner of the SLT-3, compared with the SLT-1. The brighter spots in the HAADF-STEM images in Fig. 2, for example, the centers of the smallest D clusters, suggest the positions of heavy atoms (Cr/Fe). Notably, the intensities of ten spots around the center of S in the SLT-1, and -3 are weaker than the corresponding ones of the other smallest D clusters, implying less numbers of heavy atoms in the atomic columns of the former in one periodicity along the *b* direction. The SLT-4 could be further decomposed into one D and two adhering BT structural blocks, resulting in a larger area of one BT, compared with the other SLTs. We adopted a different outline to describe the SLT-4 because the two perpendicular mirrors in it could be directly revealed, as shown in the third row in Fig. 2.

**Figure 3:**
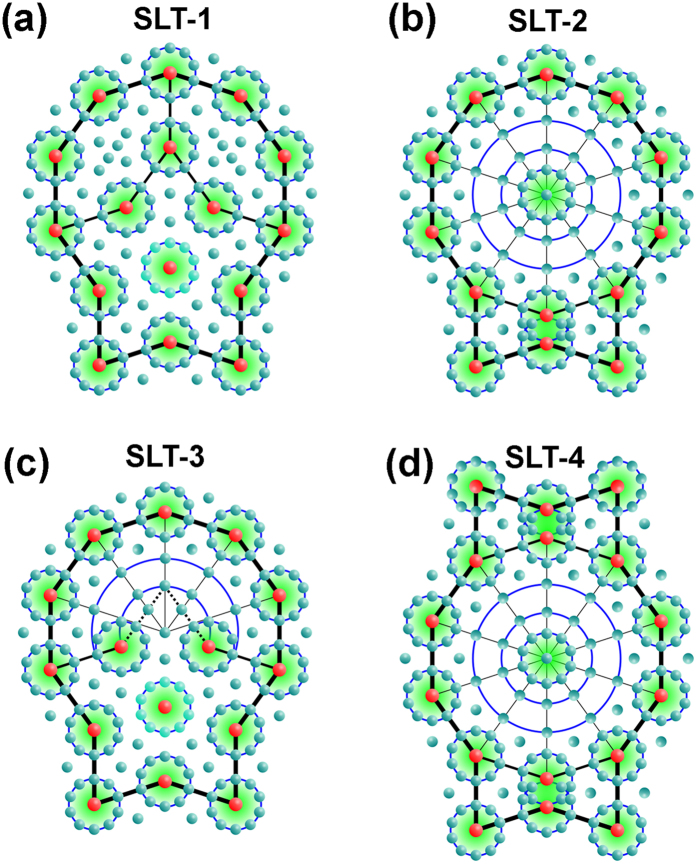
Structural schematics for each SLT. (**a**) SLT-1, (**b**) SLT-2, (**c**) SLT-3, (**d**) SLT-4. Red atoms: the transition metals (TMs) of Fe/Cr; the others: the mixed sites of Al and TM (MSs).

In Fig. 3, we deduced a structural schematic for each SLT from the images in Fig. 2. The red atoms are the transition metals (TMs) of Fe/Cr, and the others are the mixed sites of Al and TM (MSs). Furthermore, we deduced the atom positions in the inside of the H tiles of SLT-1 by comparing the H tiles in the crystal structure of orthorhombic Al_3_ Mn phase^4^ with the HAADF-STEM image in Fig. 2. However, some of atom positions in the inner of the head of SLT-3, and the inside of the largest D tile of SLT-2 and -4 are probably missed in Fig. 3b,d because of the limited resolution in Fig. 2 as well as the deficiency of known crystal structures containing such a kind of D tile. We argue that the smallest D clusters, as highlighted in gradient green in Fig. 3, could be corresponding to the icosahedral chains extending along the *b* direction because it occurs to the H tiles of Al_3_Mn phase^4^ when we analyze its crystal structure.

Unlike the often observed (3/2, 2/1) orthorhombic approximant in different alloy systems, the (2/1, 3/2) approximant is seldom observed^34^. Interestingly, the (2/1, 3/2) approximant has three additional kinds of structural tiling patterns in the Al-Cr-Fe-Si alloys than that reported previously for the same system^34^. Accordingly, these (2/1, 3/2) approximants will be denoted as (2/1, 3/2)_1_, (2/1, 3/2)_2_, (2/1, 3/2)_3_, and (2/1, 3/2)_4_, where the first three are new approximants and the last is that reported in ref. 34 (which is renamed as (2/1, 3/2)_4_ in this paper).

**Figure 4:**
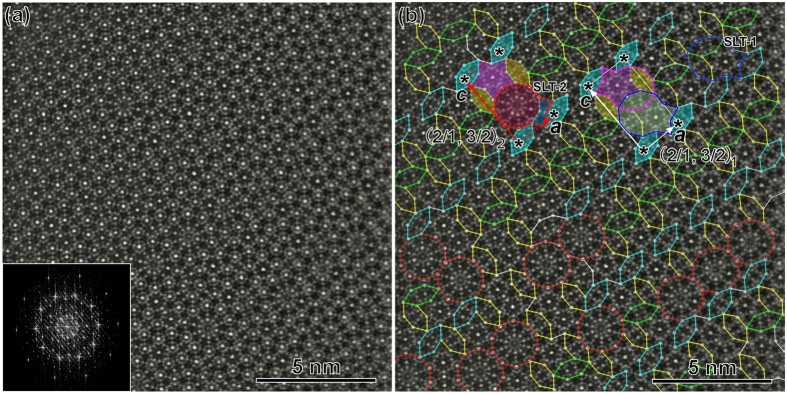
Structural tiling of (2/1, 3/2)_1_ approximant. (**a**) Atomic resolution HAADF-STEM image along the pseudo-tenfold axis of the (2/1, 3/2)_1_ approximant (from Sample 1). (**b**) Covered with structural tiles. Note that the same oriented H tiles are depicted in the same color. One unit cell of the (2/1, 3/2)_1_ and (2/1, 3/2)_2_ approximants is highlighted.

The (2/1, 3/2)_1_ approximant predominates the repeatable unit cells in Fig. 4 (from Sample 1), occasionally mixed with few (2/1, 3/2)_2_ unit cells, for example, that highlighted in the red unit cell in Fig. 4b. They possess the same dimensions, where *a* = 1.89 nm and *c* = 3.57 nm (measured from HAADF-STEM image), which are almost the same as the reported (2/1, 3/2) approximant^34^, but their crystal structures are different (will be discussed later). The strong diffraction spots in the Fourier transfer image (insert in the bottom-left corner Fig. 4a) of the original HAADF-STEM image show tenfold-symmetry, suggesting it is structurally related with the DQC. One unit cell for (2/1, 3/2)_1_ and one for (2/1, 3/2)_2_ are shown in Fig. 4b for comparison. Although the (2/1, 3/2)_1_ and (2/1, 3/2)_2_ have the same lattice parameters, the SLT-1 (in blue) in (2/1, 3/2)_1_ is replaced by the SLT-2 (in red) in (2/1, 3/2)_2_, with the others remaining the same. Accordingly, the (2/1, 3/2)_1_ and the (2/1, 3/2)_2_ approximant coexist coherently, without inducing structural strain upon each other. Accompanying the periodical unit cells, some of the substructures are arranged aperiodically, for example, the area in the bottom-left of Fig. 4b, which were often found previously in the quasicrystal-related structures of other alloys.

### (3/2, 2/1) approximants

**Figure 5:**
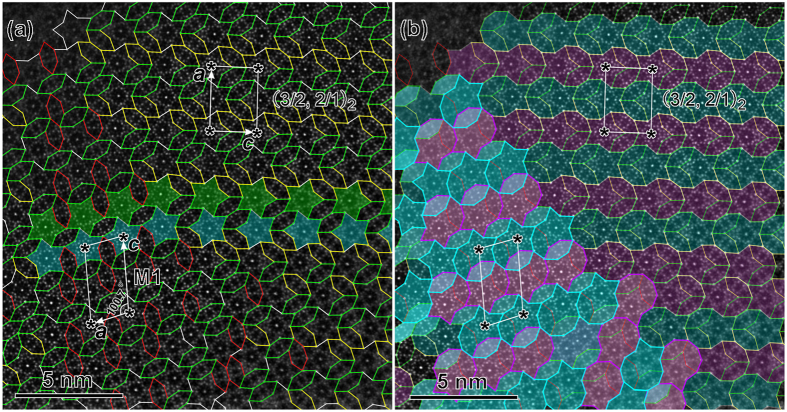
Structural tiling of (3/2, 2/1)_2_ approximant. Atomic resolution HAADF-STEM images along the *b* axis of the (3/2, 2/1)_2_ approximant (from Sample 2). (**a**) The structure is depicted using the hexagon-boat-star (HBS) tiling model, where the same oriented H tiles are drawn up in the same color. Left: monoclinic M1_ACFS_ approximant; right: (3/2, 2/1)_2_ approximant. (**b**) The structure is described by SLT blocks. The structural characteristics of the approximants and the phase boundary between them are clearly demonstrated in (**b**).

In addition to the (3/2, 2/1) approximant reported in Al-Cr-Fe-Si alloys^34^, we report here two additional (3/2, 2/1) approximants in the Al-Cr-Fe-Si alloys, with *a* = 3.04 nm, and *c* = 2.23 nm (measured from HAADF-STEM images). For clarity, we rename the reported (3/2, 2/1) approximant as (3/2, 2/1)_1_, and the two new approximants reported here as (3/2, 2/1)_2_ and (3/2, 2/1)_3_. Different from the reported (3/2, 2/1)_1_ in Al-Cr-Fe-Si alloys, where all the SLTs have the same orientation^34^, the structure of the (3/2, 2/1)_2_ approximant in the Al-Cr-Fe-Si alloys in Fig. 5b is characterized by the alternative rows of SLTs with inversed orientations, in agreement with the (3/2, 2/1) approximants found in the Al-Mn-Ni^29^. Besides the (3/2, 2/1)_2_ approximant, we note that there is one monoclinic approximant (named as M1_ACFS_ hereafter, where the subscript ACFS refers to the Al-Cr-Fe-Si system) in the left of Fig. 5a,b, which grows coherently with the former. The row of S tiles with the same orientation, as highlighted by the same color in Fig. 4a, extends from the M1_ACFS_ approximant to the (3/2, 2/1)_2_ approximant, but the direction is changed by ~18° when traversing the phase boundary. The M1_ACFS_ phase will be discussed after the orthorhombic (3/2, 2/1) approximants.

**Figure 6:**
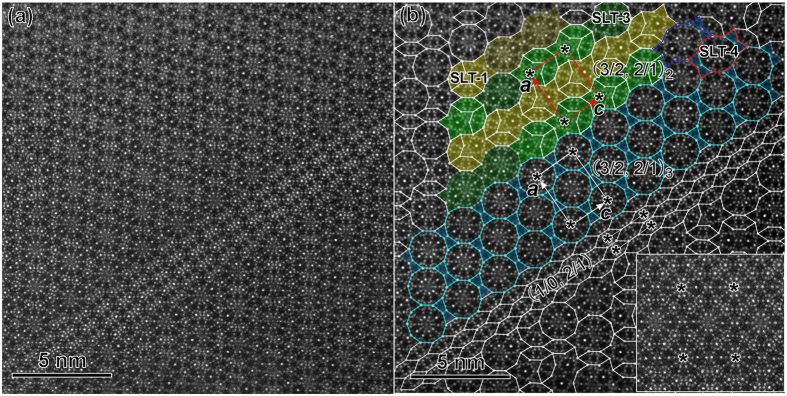
Structural tiling of (3/2, 2/1)_3_ approximant. (**a**) Atomic resolution HAADF-STEM image along the pseudo-tenfold axis of the (3/2, 2/1)_3_ approximant. (from Sample 1) (**b**) Covered with structural tiles. Note that the (3/2, 2/1)_3_ approximant, depicted in sapphire, consists of SLT-4 tiles, with one tile outlined in red in the upper-right. One enlarged (3/2, 2/1)_3_ unit cell is inserted in the bottom-right corner.

The coherent coexistence of the (3/2, 2/1)_2_ and the third (3/2, 2/1) approximant, as highlighted in sapphire and denoted as (3/2, 2/1)_3_, can be observed in Fig. 6. Note that a few of the SLT-1 tiles are replaced by the SLT-3 with the same orientation in the matrix of the (3/2, 2/1)_2_ phase, as shown in the upper-left corner of Fig. 6b, but without structural distortion to the (3/2, 2/1)_2_ owing to the same size of the SLT-1 and SLT-3. While the (3/2, 2/1)_3_ is composed of SLT-4 (the profile of one is outlined in red in the upper-right corner of Fig. 6b). The nearby SLT-4 tiles are overlapped by one BT along the *c* axis. The combined size of one D and an adhering BT in a SLT-4 is the same as that of the other types of SLT in geometry. Therefore, the (3/2, 2/1)_3_ have the same unit parameters as those of (3/2, 2/1)_1_ and (3/2, 2/1)_2_. The (3/2, 2/1)_3_ approximant not only coexists coherently with the neighboring (3/2, 2/1)_2_ and (1/0, 2/1) approximants, but also with the *a, b*, and *c* parallel to each other. The growth of the (3/2, 2/1)_3_ approximant along the *a* direction was stopped by the (3/2, 2/1)_2_ and some random structural blocks, resulting in a sandwich-like structure, where the (3/2, 2/1)_3_ approximant was clamped in-between the (3/2, 2/1)_2_ and (1/0, 2/1) approximants.

### Monoclinic approximants (*β* = 100.7°)

**Figure 7:**
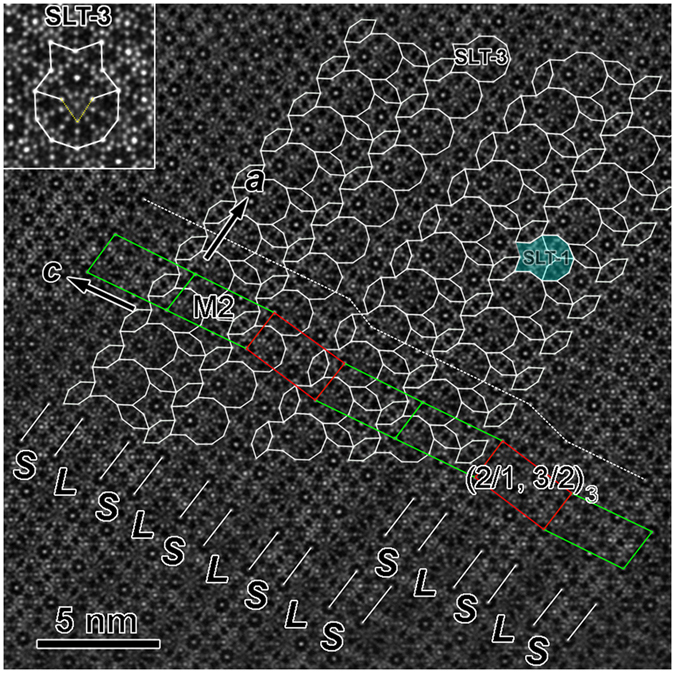
Structural tiling of M2_ACFS_ approximant. Atomic resolution HAADF-STEM image along the *b* axis of the M2_ACFS_ approximant showing an alternative coherent intergrowth of M2_ACFS_ and (2/1, 3/2)_3_ (from Sample 1). The main SLT blocks are changed to the type of SLT-3, mixed with a small amount of SLT-1. One enlarged SLT-3 is inserted in the upper-left corner.

Two monoclinic approximants with the same unit cell of *a* = 1.90 nm, *b* = 1.23 nm, *c* = 3.61 nm, and *β* = 100.7° were also found, where the *b* inherits the periodicity of the DQC. One is the M1_ACFS_ in Fig. 5, and another is the monoclinic M2_ACFS_ approximant in Fig. 7. The planar structure of M1_ACFS_ viewed along the pseudo-tenfold direction could be described by the substructures of two inversed SLT-1 tiles and an oriented H tile (Fig. 5b). The neighboring SLT-1 tiles with the same orientation are connected by sharing two sides, while the inversed SLT-1 tiles are partly overlapped by the shape of an H tile.

In comparison, the M2_ACFS_ in Fig. 7 is composed of SLT-3, S, and two-oriented H tiles. Note that the small decagonal cluster on one vertex of S in the inner of SLT-3 is missing, as marked by the dashed yellow lines in the SLT-3 inserted in the upper-left corner of Fig. 7. We refer to the S with one missing vertex as the “pseudo S”. The centers of S and pseudo S are arranged in rows, with an alternating short (S) and long (L) distance. However, the extension of the lattice of M2_ACFS_ along *c*_M2_ is disturbed, as demonstrated by the kinked dash line in Fig. 7. Consequently, one kind of (2/1, 3/2) unit cell, named as (2/1, 3/2)_3_ and highlighted by red rectangles, is observed in-between the M2_ACFS_ lattices. Therefore, the M2_ACFS_ and (2/1, 3/2)_3_ approximant grow alternatively and coherently. Furthermore, a few SLT-1 tiles are also found to be mixed within the matrix of SLT-3, for example, the SLT-1 highlighted in Fig. 7, which brings structural disorder to the matrix of M2_ACFS_.

### Geometric analysis

**Figure 8:**
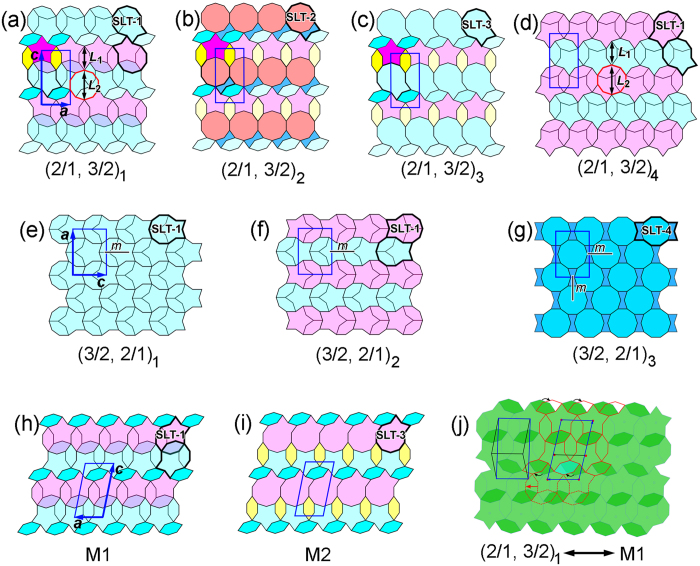
Schematic diagrams of multiple quasicrystal approximants. (**a–d**) Tiling patterns of the four kinds of (2/1, 3/2) approximants. Their lattice parameters are the same in geometry. Note the (2/1, 3/2)_3_ in (**d**) is reproduced from ref. 34. (**e–g**) Tiling patterns of the three kinds of (3/2, 2/1) approximants, where the (3/2, 2/1)_1_ are reproduced from ref. 34. Again they have the same lattice parameters. (**h–i**) Tiling patterns of the monoclinic M1_ACFS_ and M2_ACFS_ approximants. Their difference is the type of SLT, namely SLT-1 in M1_ACFS_, but SLT-3 in M2_ACFS_. (**j**) Structural relationship between (2/1, 3/2)_1_ and M1_ACFS_, where the tiles of (2/1, 3/2)_1_ are in green and those of M1_ACFS_ are in red.

We summarize the structural variants of three kinds of approximants in the schematic diagrams of Fig. 8. For simplification, the idealized structural blocks are proposed to be perfect, without distortion. Furthermore, the origins of the unit cells are set at the centers of the S or D tiles for comparison. Note that the *b* axes for all approximants inherit the periodic axis of DQC in this system, and are therefore the same as 1.23 nm.

For the four (2/1, 3/2) approximants in the first row of Fig. 8 (including the reported approximant in ref. 34, which is renamed as (2/1, 3/2)_4_), we see easily that their *a* values are equal to the diameter of the D tile (*d*_D_), deduced directly by comparing the geometry. It is also evident that the magnitude of *c*, shownin Fig. 8a–c is the same because their structural tiles are the same if we ignore the differences of the SLTs. Now let us compare the *c* values in Fig. 8a,d. The magnitude of *c* in Fig. 8a is equal to the sum of *L*_1_ + *L*_2_, where *L*_1_ and *L*_2_ are the lengths of the H tile and circumcircle diameter of the red decagon, respectively, which is the same as the *c* value in Fig. 8d. Therefore, the four (2/1, 3/2) approximants have the same unit parameters, with the *a* and *c* calculated as:


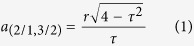



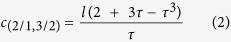


where *l* is the edge length of the structural blocks (e.g., ~0.62 nm in this paper) and the *τ* is the golden number of 0.618.

The tiling of (2/1, 3/2)_1_, (2/1, 3/2)_2_, and (2/1, 3/2)_3_ is quite similar because the part of their structures, without the SLT blocks, is exactly the same, for example, the H and S tiles highlighted by dark colors. Furthermore, the remaining part is described by an SLT tile, which also implies the similarity. The similarity of the (2/1, 3/2)_1_, (2/1, 3/2)_2_, and (2/1, 3/2)_3_ approximants might explain their coexistence in the obtained experimental images, for example, in Fig. 4.

The structural variants of (3/2, 2/1) approximants are relatively simple, as shown in the second row of Fig. 8. The magnitudes of their *a* and *c* parameters are equal, respectively, with *a* = *d*_D_ + *L*_BT_ and *c* = *d*_D_ + *W*_BT_, where *d*_D_ is the diameter of the D tile; the *L*_BT_ and *W*_BT_ are the length and the smallest width of structural block of BT, respectively. The magnitudes of *a* and *c* are calculated as:


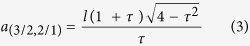



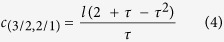


where the *l* and *τ* are the edge length of the structural blocks and the golden number of 0.618, respectively.

The structural relationship between Fig. 8e,f was discussed in our previous paper^34^, so we will not discuss this again. The SLT-4 in the (3/2, 2/1)_3_ approximant has two mirrors, which results in a *Bmm* symmetry in the (010) projection plane. Note that the SLT-4 can be further decomposed into a SLT and BT tile, where the SLT is more like the SLT-2, rather than the SLT-1 in (3/2, 2/1)_1_ and (3/2, 2/1)_2_ approximants. Although the structures of (3/2, 2/1)_1_ and (3/2, 2/1)_2_ approximants are closely related and could be deduced from each other by changing the orientations of some SLT-1 substructures^34^, the structural transformation between the (3/2, 2/1)_1_ and (3/2, 2/1)_3_ approximantsis different because the atomic structures of SLT-1 in (3/2, 2/1)_1_ and SLT-4 in (3/2, 2/1)_3_ are different.

Analogous to the structural difference among (2/1, 3/2)_1_, (2/1, 3/2)_2_, and (2/1, 3/2)_3_, the difference between the M1_ACFS_ and M2_ACFS_ is also caused by the different SLTs, namely the SLT-1 in M1_ACFS_ and the SLT-3 in M2_ACFS_. The other part of these two monoclinic phases is exactly the same. Therefore, the two monoclinic phases of M1_ACFS_ and M2_ACFS_ have the same lattice parameters.

We note that the (2/1, 3/2)_1_ approximant in Fig. 8a and the monoclinic M1_ACFS_ approximant in Fig. 8h have the same magnitude of *a*, and also the same tiles of H and SLT-1. Therefore, their structural relationship is compared in Fig. 8j, where the (2/1, 3/2)_1_ is in green and viewed from an inverse direction with respect to that in Fig. 8a, to obtain SLT blocks with the same orientation as those in Fig. 8h. Meanwhile, the monoclinic M1_ACFS_ approximant is depicted by red lines and superposed onto the (2/1, 3/2)_1_ approximant (Fig. 8j). The upper SLTs (solid lines) in both phases are completely overlapped. However, the lower SLTs of M1_ACFS_ (dash lines) shift a distance of the width of the H tile (~0.74 nm) along *a*_M1_ (marked by the red arrow) with respect to the corresponding tiles in the (2/1, 3/2)_1_ approximant. Accordingly, the lower parallelogram formed by connecting the centers of the S tiles in the (2/1, 3/2)_1_ approximant is changed to the position where the short edges nearly line up with the corresponding edges of the upper parallelogram in the M1_ACFS_ approximant. Furthermore, some of the H tiles also change their positions and orientations, as marked by the curved arrows, to fill the space in-between the SLTs of the M1_ACFS_ approximant, for example, the H tile filled in sky blue.

We summarize the lattice parameters of the approximants mentioned above in Table 1. Moreover, the plane crystallographic groups in the (*a, c*) plan of these approximants are also listed after analyzing the geometry in Fig. 8. We emphasize that the (2/1, 3/2)_1_, (2/1, 3/2)_2_, and (2/1, 3/2)_3_ approximants should be ascribed to monoclinic phases from the viewpoint of symmetry because neither *p*1 nor *p*2 plane crystallographic group exists for an orthorhombic phase although a rectangle lattice could be drawn in Fig. 8a–c, which has also been noted for the Al_71_ Ni_22_ Co_7_ approximant by Abe *et al*.^22^.

## Conclusion

By means of atomic resolution HAADF-STEM images, we have found three types of DQC approximants in Al-Cr-Fe-Si systems, where each type has several structural variants with the same lattice parameters, but differ in their crystal structures. The (2/1, 3/2)-type approximants contain four structural variants, in which the (2/1, 3/2)_1_, (2/1, 3/2)_2_, and (2/1, 3/2)_3_ approximants belong to monoclinic phases by considering the symmetries although the *β* = 90°. The orthorhombic (3/2, 2/1)-type approximant includes three structural variants. Furthermore, we also found two more monoclinic approximants (M1_ACFS_, and M2_ACFS_) with the same unit cell: *a* = 1.90 nm, *b* = 1.23 nm, *c* = 3.61 nm, and *β* = 100.7°, but varied in their crystal structures owing to the different SLTs. The structural variations for each type are closely related with changeable SLTs, which are further classified into four types: SLT-1, SLT-2, SLT-3, and SLT-4. The types, orientations, and connections of the structural blocks, especially the SLTs, are responsible for the multiplicity of the approximants with the same unit cell reported here.

## Experimental

Approximately 1 Kg of the master Al-Cr-Fe-Si alloy with a nominal composition of Al_60_ Cr_20_ Fe_10_ Si_10_ was prepared by melting high-purity elements in an induction furnace under vacuum. The samples investigated in this study were treated according to the following process: several pieces of the master Al_60_ Cr_20_ Fe_10_ Si_10_ alloy were first heated at 1070 °C for 24 h in an evacuated quartz tube, and then cooled slowly to 1000 °C for 24 h, and then followed by cooling in the furnace (Sample 1). Part of the sample after the heat treatment was then annealed at 900 °C for 15 days in a vacuum, and then cooled in the furnace by shutting off the power (Sample 2). Powder samples were adopted for TEM observations. An FEI Tecnai F30 transmission electron microscope equipped with an energy dispersive X-ray spectrometer (EDS) was first used to check the phases and the composition. A JEM-ARM200F transmission electron microscope equipped with a Cs-probe corrector and Cs-image corrector was used to obtain HAADF-STEM images at an atomic resolution. The inner and outer acceptance semi-angles for HAADF-STEM imaging were 90 and 370 mrad, respectively.

## Additional Information

**How to cite this article**: He, Z. *et al*. Multiple quasicrystal approximants with the same lattice parameters in Al-Cr-Fe-Si alloys. *Sci. Rep.* **7**, 40510; doi: 10.1038/srep40510 (2017).

**Publisher's note:** Springer Nature remains neutral with regard to jurisdictional claims in published maps and institutional affiliations.

## Figures and Tables

**Table 1:** Approximants of DQC in Al-Cr-Fe-Si system.

Approximants	Plane crystallographic group in (*a, c*)	Lattice parameters*		
		*a* (nm)	*c* (nm)	*β* (°)
(2/1, 3/2)_1_	*p*2	1.89	3.57	90
(2/1, 3/2)_2_	*p*1			
(2/1, 3/2)_3_	*p*1			
(2/1, 3/2)_4_	*pg (g*⊥*a*)			
(3/2, 2/1)_1_	*cm*	3.04	2.23	90
(3/2, 2/1)_2_	*pmg (g*⊥*c*)			
(3/2, 2/1)_3_	*cmm*			
M1	*p*2	1.90	3.61	100.7
M2	*p*1			

^*^Measured from HAADF-STEM images. All these approximants have the same *b* ≈ 1.23 nm.
